# Cadmium sulfite hexahydrate revisited

**DOI:** 10.1107/S1600536808011409

**Published:** 2008-06-07

**Authors:** Sergio Baggio, Andrés Ibáñez, Ricardo Baggio

**Affiliations:** a Universidad Nacional de la Patagonia, Sede Puerto Madryn, and, CenPat, CONICET, 9120 Puerto Madryn, Chubut, Argentina; b Departamento de Física, Facultad de Ciencias Físicas y Matemáticas, Universidad de Chile and CIMAT, Casilla 487-3, Santiago de Chile, Chile; c Departamento de Física, Comisión Nacional de Energía Atómica, Buenos Aires, Argentina

## Abstract

The present structural revision of the title compound, tetra­cadmium tetra­sulfite hexa­hydrate, [Cd_4_(SO_3_)_4_(H_2_O)_5_]·H_2_O, is a low-temperature upgrade (*T* = 100 K and *R* = 0.017) of the original room-temperature structure reported by Kiers & Vos [*Cryst. Struct. Commun.* (1978). **7**, 399–403; *T* = 293 K and *R* = 0.080). The compound is a three-dimensional polymer with four independent cadmium centres, four sulfite anions and six water mol­ecules, five of them coordinated to two cadmium centres and the remaining one an unbound solvent mol­ecule which completes the asymmetric unit. There are two types of cadmium environment: CdO_8_ (through four chelating sulfite ligands) and CdO_6_ (by way of six monocoordinated ligands). The former groups form planar arrays [parallel to (001) and separated by half a unit cell translation along *c*], made up of chains running along [110] and [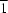
10], respectively. These chains are, in turn, inter­connected both in an intra­planar as well as in an inter­planar fashion by the latter CdO_6_ polyhedra into a tight three-dimensional framework. There is, in addition, an extensive network of hydrogen bonds, in which all 12 water H atoms act as donors and eight O atoms from all four sulfite groups and two water mol­ecules act as acceptors.

## Related literature

For related literature, see: Agre *et al.* (1981 ▶); Brown & Altermatt (1985 ▶); Elder *et al.* (1978 ▶); Harvey *et al.* (2006 ▶); Kiers & Vos (1978 ▶); Larsson & Kierkegaard (1969 ▶).

## Experimental

### 

#### Crystal data

[Cd_4_(SO_3_)_4_(H_2_O)_5_]·H_2_O
*M* *_r_* = 877.94
Monoclinic, 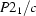

*a* = 12.1406 (3) Å
*b* = 10.5485 (3) Å
*c* = 13.9329 (4) Å
β = 103.93 (1)°
*V* = 1731.82 (11) Å^3^
*Z* = 4
Mo *K*α radiation
μ = 5.41 mm^−1^
*T* = 150 (2) K
0.24 × 0.12 × 0.08 mm

#### Data collection

Bruker SMART CCD area-detector diffractometer
Absorption correction: multi-scan (*SADABS*; Sheldrick, 2001 ▶) *T*_min_ = 0.40, *T*_max_ = 0.64
31336 measured reflections
3959 independent reflections
3922 reflections with *I* > 2σ(*I*)
*R*_int_ = 0.021

#### Refinement

*R*[*F* ^2^ > 2σ(*F* ^2^)] = 0.017
*wR*(*F* ^2^) = 0.040
*S* = 1.26
3959 reflections
284 parameters
18 restraints
All H-atom parameters refined
Δρ_max_ = 0.70 e Å^−3^
Δρ_min_ = −0.56 e Å^−3^

### 

Data collection: *SMART* (Bruker, 2001 ▶); cell refinement: *SAINT* (Bruker, 2001 ▶); data reduction: *SAINT*; program(s) used to solve structure: *SHELXS97* (Sheldrick, 2008 ▶); program(s) used to refine structure: *SHELXL97* (Sheldrick, 2008 ▶); molecular graphics: *SHELXTL* (Sheldrick, 2008 ▶); software used to prepare material for publication: *SHELXTL* and *PLATON* (Spek, 2003 ▶).

## Supplementary Material

Crystal structure: contains datablocks global, I. DOI: 10.1107/S1600536808011409/br2070sup1.cif

Structure factors: contains datablocks I. DOI: 10.1107/S1600536808011409/br2070Isup2.hkl

Additional supplementary materials:  crystallographic information; 3D view; checkCIF report

## Figures and Tables

**Table 1:** Selected bond lengths (Å)

Cd1—O13	2.2452 (18)
Cd1—O32^i^	2.2839 (18)
Cd1—O22	2.3065 (18)
Cd1—O21	2.4078 (17)
Cd1—O12	2.4542 (18)
Cd1—O31	2.4752 (18)
Cd1—O23	2.6544 (18)
Cd1—O12^i^	2.7665 (19)
Cd2—O34	2.3311 (18)
Cd2—O14^ii^	2.3365 (18)
Cd2—O33	2.3440 (18)
Cd2—O11	2.3545 (18)
Cd2—O24^ii^	2.4074 (18)
Cd2—O23	2.4446 (18)
Cd2—O14	2.6091 (18)
Cd2—O21	2.8126 (18)
Cd3—O2*W*	2.215 (2)
Cd3—O1*W*	2.2272 (19)
Cd3—O3*W*	2.278 (2)
Cd3—O31	2.3201 (17)
Cd3—O12	2.3482 (18)
Cd3—O11^iii^	2.3518 (18)
Cd4—O34	2.2412 (18)
Cd4—O4*W*	2.2599 (18)
Cd4—O5*W*	2.2601 (19)
Cd4—O32^iv^	2.2816 (18)
Cd4—O23^v^	2.3203 (17)
Cd4—O21	2.3571 (17)

Symmetry codes: (i) 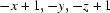; (ii) 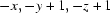; (iii) 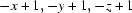; (iv) 
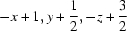
; (v) 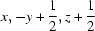.

**Table 2:** Hydrogen-bond geometry (Å, °)

*D*—H⋯*A*	*D*—H	H⋯*A*	*D*⋯*A*	*D*—H⋯*A*
O1*W*—H1*WA*⋯O13^i^	0.82 (3)	1.88 (2)	2.693 (3)	170 (4)
O1*W*—H1*WB*⋯O14^vi^	0.82 (3)	1.93 (2)	2.679 (3)	151 (3)
O2*W*—H2*WA*⋯O4*W*^iv^	0.82 (3)	1.93 (2)	2.719 (3)	162 (4)
O2*W*—H2*WB*⋯O6*W*	0.82 (3)	1.95 (2)	2.681 (3)	149 (4)
O3*W*—H3*WA*⋯O6*W*^vii^	0.82 (3)	2.02 (2)	2.833 (3)	172 (4)
O3*W*—H3*WB*⋯O22^i^	0.82 (3)	2.29 (2)	3.092 (3)	168 (4)
O4*W*—H4*WA*⋯O22	0.82 (3)	1.87 (2)	2.651 (3)	159 (3)
O4*W*—H4*WB*⋯O31^v^	0.82 (3)	1.98 (2)	2.780 (3)	167 (3)
O5*W*—H5*WA*⋯O33	0.82 (3)	2.05 (2)	2.848 (3)	167 (4)
O5*W*—H5*WB*⋯O24^viii^	0.82 (3)	1.92 (2)	2.708 (3)	162 (4)
O6*W*—H6*WA*⋯O24^ix^	0.82 (3)	2.16 (2)	2.876 (3)	146 (4)
O6*W*—H6*WB*⋯O33^x^	0.82 (3)	2.18 (2)	2.948 (3)	157 (4)

Symmetry codes: (i) 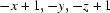; (iv) 
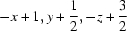
; (v) 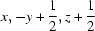; (vi) 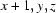; (vii) 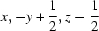; (viii) 
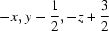
; (ix) 
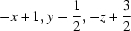
; (x) 
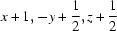
.

**Fig. 1.**
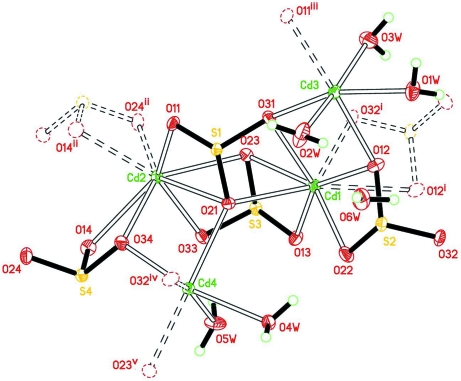
a view of the structure with the independent atoms drawn in full 50% displacement ellipsoids. The symmetry related part, in dashed ellipsoids and bonds. Hydrogen interactions not shown, for clarity. Symmetry codes: (i) -x + 1, -y, -z + 1; (ii) -x, -y + 1, -z + 1; (iii) -x + 1, -y + 1, -z + 1; (iv) -x + 1, y + 1/2, -z + 3/2; (v) x, -y + 1/2, z + 1/2.

**Fig. 2.**
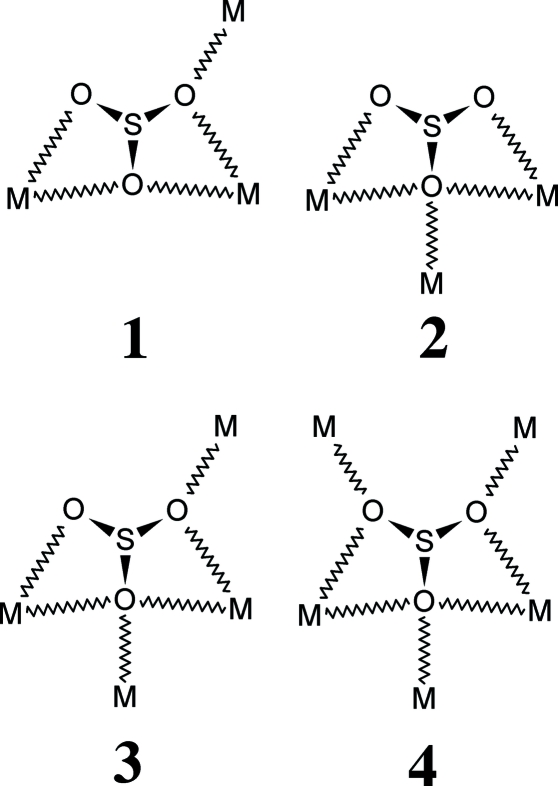
The the coordination of the anions anions through all their three donor O atoms in µ3, µ4 and µ5 modes.

**Fig. 3.**
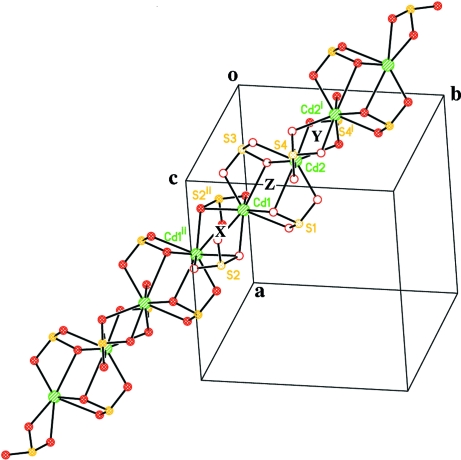
A detailed view of one of the two Cd1—Cd2 chains (the one evolving along [110] and embedding symmetry centers at [1/2,0, 1/2] (X) and [0, 1/2, 1/2] (Y). Note the local pseudo symmetry centre Z.

**Fig. 4.**
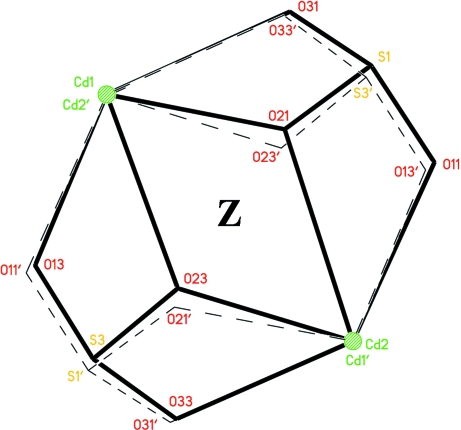
Least-squares overlap of the Cd1—Cd2 nucleus with its inverted image thorugh a (X=0.255, Y=0.263, Z=0.500, site Z) inversion.

**Fig. 5.**
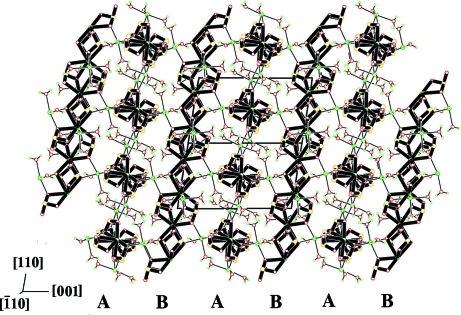
Schematic representation of the structure packing. See text for details.

## supplementary crystallographic information

### Comment

The sulfite SO_3_^-2^ ion is a most versatile inorganic ligand: the four atoms in the group can act as coordination donors and thus the molecule displays an enormous collection of different binding modes, from the very simple µ-S, as in pentaamminesulfite cobalt(III) chloride hydrate (Elder *et al.*, 1978), or µ-O, as in trisodium ethylenediamine-tetra-acetato-sulfite-indium(iii) tetrahydrate (Agre *et al.*, 1981), to an impressive µ_10_-S:*O*:*O*:*O*:*O*':*O*':*O*':*O*":*O*":*O*" in anhydrous disodium sulfite (Larsson & Kierkegaard, 1969).

In combination with transition metals the anion can generate interesting structures, many of them reported in pioneering structural works. Some of these, however, even if proficiently worked out to the state of the art at the time of publication, appear nowadays below acceptable (and desirable) standards. This was the case for cadmium sulfite hydrate [Cd_4_(SO_3_)_4_(H_2_O)_5_.(H_2_O)], originally reported by Kiers & Vos, 1978, in a R.T.,low resolution structure determination (T: 293 K, *R*:0.080) and for which we present herein an upgrade, by way of a low temperature data refinement (T: 100 K, *R*: 0.017).

The structure (shown in Fig. 1) is a three-dimensional polymer with four independent cadmium centres, four sulfite anions and six water molecules, five of them coordinated to two cadmium centres and the remaining one, an unbound solvate which completes the asymmetric unit.

The cadmium environments in the structure split naturally into two types, *viz*.: two CdO_8_, centred at Cd1 and Cd2 and achieved through four chelating sulfite bites each, and two CdO_6_, centred at Cd3 and Cd4 and where no chelating bites whatsoever take part, the donor O atoms being either bridging (sulfite) or monocoordinated (aqua) (Table 1).

The two octacoordinated cadmium centres are comparable, but due to multiple chelation the corresponding CdO_8_ polyhedra are difficult to describe by any regular model. However, both centres present a similar "tetrahedral" environment of ligands, the one around Cd1 being more flattened and describable as something midway a tetrahedral and a square planar arrangement. The one around Cd2, instead, is much more biased towards a tetrahedral shape.

In this regard the groups are adequate for a Vector Bond Valence treatment (hereafter VBV, Harvey *et al.*, 2006), a novel approach tending to a simpler description of multidentate binding, in which the action of each ligand is replaced by a single interaction vector, the Vector Bond Valence (or VBV), derived from the individual bond valences (Brown & Altermatt, 1985) of the coordinating atoms.

Even though for the four-ligand coordination geometry the VBV model would not predict a definite geometry for the four VBV vectors, the requirement of a bond valence of ~2 for both cations and a nil resultant of their vectorial sum would still be in force.

These requirements are satisfactorily fulfilled in both cases, with a scalar Bond Valence of 2.017 and 1.949, and a resultant VBV of 0.047 and 0.084 valence units for Cd1 and Cd2, respectively. Also the geometries of the (distorted) tetrahedra are correctly described by the VBV vectors, with the flattened Cd1 polyhedron presenting two large angles between *trans* VBV (126.8 (1) and 130.8 (1)°), and a much tighter span for the rest (Range: 94.9 (1)–111.7 (1)°) while the tetrahedron centred at Cd2 presents a close angle distribution throughout (Range: 100.9 (1)–115.9 (1)°).

The remaining cadmium centres Cd3 and Cd4, lacking any chelating ligand in their polyhedra, present rather regular octahedral arrangements (Table 1).

The anions coordinate through all their three donor O atoms, though not through sulfur, in µ_3_, µ_4_ and µ_5_ modes (Fig. 2). The internal geometry of the anions is quite regular and similar, as judged by the S—O (Å), O—S—O (°) mean values: S1, 1.543 (7), 103.2 (21); S2, 1.536 (6), 102.5 (12); S3, 1.540 (19), 103.3 (24); S4, 1.539 (18), 102.8 (17).

In addition to the diversity in cation environments, there is a more profound difference setting apart these two types of polyhedra, and it consists in their quite diverse structural function.

On one side, both CdO_8_ groups join to each other forming two sets of straight chains (See Fig. 3 for details) at *z* = 0, running along [110], and *z* = 1/2, running along [110], (A and B in Fig. 5; see below) both orientations subtending and angle of 98.0 (1)° to each other. Inspection of Fig. 3 reveals that the chains embed the crystallographic symmetry centres at sites *X* (at 1/2,0,1/2) and Y (at 0,1/2,1/2). There is, however, an extra, nearly perfect (though non crystallographic) pseudo centre midway the former two at site *Z* = 0.255,0.263, 1/2, relating Cd1 with Cd2, and SO_3_(1) with SO_3_(3). The degree of local pseudo symmetry involved can be assessed by the least squares fit of the Cd1, Cd2, SO_3_(1) and SO_3_(3) group (built up around the pseudo centre) and its inverted image, which gives a mean deviation of 0.11 (1)Å and a maximum of 0.14 (1)Å for the O31—O33 pair (Fig. 4). Fig. 5 shows the way in which these one-dimensional structures interact with each other: the chains containing Cd1—Cd2 (in bold) appear at nearly right angles to each other, either coming out the plane of the paper (type A) or lying on the plane of the paper (type B). The remaining polyhedra, in weak lining, interconnect them in such a way that while Cd3O_6_ groups link parallel chains (A—A, B—B) along [110] and [110], Cd4O_6_ ones link perpendicular chains (A—B), along [001], with the final result of a very tight three-dimensional framework building up.

All six water molecules in the structure are involved in H-bonding through their twelve H atoms as donors (Table 2). The acceptor role is covered by eight O atoms coming from the four sulfite groups and two water molecules. The sulfite anions participate in a rather uneven way, *e.g.*: sulfite(1) through only one H-bond involving O31, sulfite(2), through two bonds, both involving O22, sulfite(3) and sulfite(4) through three bonds each, *via* O13 and O33 (twice) for the former, and by way of O14 and O24 (twice) for the latter. Among the water molecules, only one aqua participates as an acceptor (O4W, bound to Cd4), the remaining one being the crystal water O6W, which receives two bonds, and thus completes the scheme.

Contrasting with what is found in other sulfite structures, strong involvement in H-bonding of sulfite O atoms does not seem to weaken their S—O interactions; thus, the three O's which receive two H-bonds each and could thus be suspected of being affected by a strong electron-withdrawal effect, irrespective of this fact present either similar or significantly shorter S—O distances in their SO_3_ groups. This can be assesed in the following data, where the S—O under consideration, its bond length, and the mean value of the remaining two S—O's in the group (mean-rest) are shown. Thus, in sulfite(2), S2—O22: 1.5302 (19), mean-rest: 1.541 (2) Å; in sulfite(3), S3—O33: 1.5269 (18), mean-rest: 1.547 (15) Å; in sulfite(4), S4—O24: 1.5189 (19), mean-rest: 1.549 (6) Å.

The complexity of this H-bonding scheme turns almost impossible any meaningful representation of the network to which it gives rise, for which a detailed packing figure including them has been spared, for the sake of clarity.

### Experimental

The compound was obtained by slow inter diffusion of Na_2_SO_3_ and Cd(CH_3_CO_2_)_2_ aqueous solutions in (1:1) molar ratio. The connecting path between the two vessels was filled with an aqueous solution of NaCH_3_CO_2_, in order to minimize concentration gradients. After several weeks of unperturbed diffusion a crop of colourless, prismatic crystals of the title compound was obtained.

### Refinement

Hydrogen atoms (all of them pertaining to water molecules) were found in the difference- Fourier synthesis and refined with restrained O—H:0.82 (3) Å, H···H:1.35 (3) Å and free isotropic displacement parameters.

### Crystal data

**Table:**

[Cd_4_(SO_3_)_4_(H_2_O)_5_]·H_2_O	*F*_000_ = 1648
*M**_r_* = 877.94	*D*_x_ = 3.367 Mg m^−^^3^
Monoclinic, *P*2_1_/*c*	Mo *K*α radiation λ = 0.71073 Å
Hall symbol: -P 2ybc	Cell parameters from 9999 reflections
*a* = 12.1406 (3) Å	θ = 1.9–27.2º
*b* = 10.5485 (3) Å	µ = 5.41 mm^−^^1^
*c* = 13.9329 (4) Å	*T* = 150 (2) K
β = 103.93 (1)º	Prisms, colourless
*V* = 1731.82 (11) Å^3^	0.24 × 0.12 × 0.08 mm
*Z* = 4	

### Data collection

**Table:**

Bruker CCD area-detector diffractometer	3959 independent reflections
Radiation source: fine-focus sealed tube	3922 reflections with *I* > 2σ(*I*)
Monochromator: graphite	*R*_int_ = 0.021
*T* = 150(2) K	θ_max_ = 27.8º
φ and ω scans	θ_min_ = 1.7º
Absorption correction: multi-scan(SADABS; Sheldrick, 2001)	*h* = −15→15
*T*_min_ = 0.40, *T*_max_ = 0.64	*k* = −13→13
31336 measured reflections	*l* = −18→18

### Refinement

**Table:**

Refinement on *F*^2^	Secondary atom site location: difference Fourier map
Least-squares matrix: full	Hydrogen site location: inferred from neighbouring sites
*R*[*F*^2^ > 2σ(*F*^2^)] = 0.017	All H-atom parameters refined
*wR*(*F*^2^) = 0.040	*w* = 1/[σ^2^(*F*_o_^2^) + (0.0143*P*)^2^ + 2.6649*P*] where *P* = (*F*_o_^2^ + 2*F*_c_^2^)/3
*S* = 1.26	(Δ/σ)_max_ = 0.001
3959 reflections	Δρ_max_ = 0.70 e Å^−^^3^
284 parameters	Δρ_min_ = −0.56 e Å^−^^3^
18 restraints	Extinction correction: SHELXL97 (Sheldrick, 2008), Fc^*^=kFc[1+0.001xFc^2^λ^3^/sin(2θ)]^-1/4^
Primary atom site location: structure-invariant direct methods	Extinction coefficient: 0.00228 (6)

### Special details

**Table:**

Geometry. All e.s.d.'s (except the e.s.d. in the dihedral angle between two l.s. planes) are estimated using the full covariance matrix. The cell e.s.d.'s are taken into account individually in the estimation of e.s.d.'s in distances, angles and torsion angles; correlations between e.s.d.'s in cell parameters are only used when they are defined by crystal symmetry. An approximate (isotropic) treatment of cell e.s.d.'s is used for estimating e.s.d.'s involving l.s. planes.

### Fractional atomic coordinates and isotropic or equivalent isotropic displacement parameters (Å^2^)

**Table:**

	*x*	*y*	*z*	*U*_iso_*/*U*_eq_	
Cd1	0.374818 (15)	0.126886 (17)	0.512881 (12)	0.01034 (5)	
Cd2	0.126635 (15)	0.392254 (17)	0.488405 (12)	0.00985 (5)	
Cd3	0.651371 (15)	0.304514 (17)	0.535886 (13)	0.01196 (5)	
Cd4	0.251030 (14)	0.282648 (17)	0.748125 (12)	0.00938 (5)	
S1	0.39723 (5)	0.40991 (6)	0.57849 (4)	0.00932 (11)	
O11	0.30501 (15)	0.49090 (17)	0.51101 (13)	0.0124 (4)	
O21	0.33019 (15)	0.29972 (16)	0.60990 (13)	0.0126 (4)	
O31	0.45766 (15)	0.34041 (17)	0.50750 (13)	0.0126 (3)	
S2	0.58417 (5)	0.04129 (6)	0.66523 (4)	0.01119 (12)	
O12	0.58184 (16)	0.10397 (17)	0.56483 (13)	0.0151 (4)	
O22	0.45763 (15)	0.03386 (19)	0.66270 (13)	0.0167 (4)	
O32	0.61638 (16)	−0.09653 (17)	0.64740 (13)	0.0139 (4)	
S3	0.11611 (5)	0.11583 (6)	0.42209 (4)	0.01077 (12)	
O13	0.20348 (15)	0.03728 (17)	0.49607 (13)	0.0142 (4)	
O23	0.19010 (15)	0.22405 (17)	0.39362 (13)	0.0131 (4)	
O33	0.04828 (15)	0.18855 (17)	0.48327 (13)	0.0146 (4)	
S4	0.01096 (5)	0.43144 (6)	0.66480 (4)	0.01148 (12)	
O14	−0.05477 (15)	0.40529 (17)	0.55726 (13)	0.0144 (4)	
O24	−0.01213 (16)	0.57150 (18)	0.67606 (13)	0.0166 (4)	
O34	0.13592 (15)	0.42562 (18)	0.65556 (13)	0.0137 (4)	
O1W	0.81699 (17)	0.20277 (19)	0.57164 (16)	0.0197 (4)	
H1WA	0.814 (3)	0.1330 (18)	0.546 (3)	0.045 (12)*	
H1WB	0.868 (2)	0.246 (3)	0.559 (3)	0.030 (10)*	
O2W	0.7011 (2)	0.3753 (2)	0.68995 (16)	0.0286 (5)	
H2WA	0.693 (3)	0.4504 (13)	0.700 (3)	0.045 (12)*	
H2WB	0.760 (2)	0.347 (3)	0.725 (2)	0.037 (11)*	
O3W	0.61491 (18)	0.2464 (2)	0.37382 (15)	0.0203 (4)	
H3WA	0.6757 (19)	0.249 (4)	0.358 (3)	0.046 (12)*	
H3WB	0.586 (3)	0.1767 (18)	0.359 (3)	0.034 (11)*	
O4W	0.36903 (15)	0.11969 (18)	0.80645 (13)	0.0133 (4)	
H4WA	0.411 (2)	0.101 (4)	0.7709 (19)	0.032 (11)*	
H4WB	0.405 (2)	0.133 (4)	0.8633 (11)	0.034 (11)*	
O5W	0.12980 (17)	0.1237 (2)	0.68645 (14)	0.0186 (4)	
H5WA	0.100 (3)	0.132 (4)	0.6275 (9)	0.033 (10)*	
H5WB	0.082 (2)	0.113 (4)	0.718 (2)	0.047 (13)*	
O6W	0.83535 (18)	0.2464 (2)	0.83907 (16)	0.0233 (4)	
H6WA	0.861 (3)	0.180 (2)	0.822 (3)	0.058 (15)*	
H6WB	0.885 (3)	0.284 (3)	0.879 (3)	0.052 (14)*	

### Atomic displacement parameters (Å^2^)

**Table:**

	*U*^11^	*U*^22^	*U*^33^	*U*^12^	*U*^13^	*U*^23^
Cd1	0.00977 (9)	0.01060 (9)	0.01059 (9)	0.00073 (6)	0.00235 (7)	0.00000 (6)
Cd2	0.00950 (9)	0.00952 (9)	0.01055 (9)	0.00085 (6)	0.00243 (6)	0.00000 (6)
Cd3	0.01045 (9)	0.01000 (9)	0.01560 (9)	0.00038 (6)	0.00346 (7)	0.00120 (6)
Cd4	0.00934 (9)	0.00987 (9)	0.00873 (9)	0.00011 (6)	0.00176 (7)	0.00026 (6)
S1	0.0086 (3)	0.0090 (3)	0.0101 (3)	0.0010 (2)	0.0019 (2)	0.0000 (2)
O11	0.0097 (8)	0.0102 (8)	0.0169 (9)	0.0021 (7)	0.0027 (7)	0.0033 (7)
O21	0.0167 (9)	0.0100 (8)	0.0131 (8)	0.0000 (7)	0.0071 (7)	0.0011 (6)
O31	0.0110 (8)	0.0147 (9)	0.0130 (8)	0.0020 (7)	0.0047 (7)	0.0004 (7)
S2	0.0119 (3)	0.0101 (3)	0.0110 (3)	0.0023 (2)	0.0015 (2)	−0.0009 (2)
O12	0.0159 (9)	0.0134 (9)	0.0150 (9)	−0.0016 (7)	0.0019 (7)	0.0036 (7)
O22	0.0130 (9)	0.0238 (10)	0.0145 (9)	0.0067 (8)	0.0054 (7)	0.0030 (7)
O32	0.0172 (9)	0.0101 (8)	0.0136 (8)	0.0053 (7)	0.0022 (7)	0.0003 (7)
S3	0.0099 (3)	0.0109 (3)	0.0115 (3)	0.0002 (2)	0.0025 (2)	−0.0015 (2)
O13	0.0128 (9)	0.0107 (9)	0.0186 (9)	0.0004 (7)	0.0029 (7)	0.0019 (7)
O23	0.0143 (9)	0.0129 (9)	0.0135 (8)	0.0006 (7)	0.0060 (7)	0.0003 (7)
O33	0.0134 (9)	0.0148 (9)	0.0179 (9)	0.0012 (7)	0.0082 (7)	−0.0013 (7)
S4	0.0106 (3)	0.0121 (3)	0.0123 (3)	0.0021 (2)	0.0038 (2)	0.0015 (2)
O14	0.0138 (9)	0.0145 (9)	0.0139 (8)	−0.0008 (7)	0.0010 (7)	−0.0009 (7)
O24	0.0201 (10)	0.0140 (9)	0.0164 (9)	0.0065 (8)	0.0057 (7)	−0.0001 (7)
O34	0.0096 (8)	0.0172 (9)	0.0144 (8)	0.0029 (7)	0.0033 (7)	0.0025 (7)
O1W	0.0131 (10)	0.0123 (9)	0.0336 (11)	−0.0006 (8)	0.0052 (8)	−0.0019 (8)
O2W	0.0419 (14)	0.0186 (11)	0.0201 (10)	0.0122 (10)	−0.0027 (9)	−0.0021 (8)
O3W	0.0221 (11)	0.0204 (10)	0.0206 (10)	−0.0038 (8)	0.0094 (8)	−0.0028 (8)
O4W	0.0134 (9)	0.0144 (9)	0.0119 (8)	0.0023 (7)	0.0028 (7)	−0.0005 (7)
O5W	0.0201 (10)	0.0231 (10)	0.0129 (9)	−0.0100 (8)	0.0046 (8)	−0.0013 (8)
O6W	0.0177 (10)	0.0271 (12)	0.0230 (10)	0.0056 (9)	0.0006 (8)	−0.0002 (9)

### Geometric parameters (Å, °)

**Table:**

Cd1—O13	2.2452 (18)	Cd3—O12	2.3482 (18)
Cd1—O32^i^	2.2839 (18)	Cd3—O11^iii^	2.3518 (18)
Cd1—O22	2.3065 (18)	Cd4—O34	2.2412 (18)
Cd1—O21	2.4078 (17)	Cd4—O4W	2.2599 (18)
Cd1—O12	2.4542 (18)	Cd4—O5W	2.2601 (19)
Cd1—O31	2.4752 (18)	Cd4—O32^iv^	2.2816 (18)
Cd1—O23	2.6544 (18)	Cd4—O23^v^	2.3203 (17)
Cd1—O12^i^	2.7665 (19)	Cd4—O21	2.3571 (17)
Cd2—O34	2.3311 (18)	S1—O11	1.5364 (18)
Cd2—O14^ii^	2.3365 (18)	S1—O21	1.5416 (18)
Cd2—O33	2.3440 (18)	S1—O31	1.5504 (18)
Cd2—O11	2.3545 (18)	S2—O22	1.5302 (19)
Cd2—O24^ii^	2.4074 (18)	S2—O32	1.5410 (18)
Cd2—O23	2.4446 (18)	S2—O12	1.5413 (18)
Cd2—O14	2.6091 (18)	S3—O33	1.5269 (18)
Cd2—O21	2.8126 (18)	S3—O13	1.5323 (18)
Cd3—O2W	2.215 (2)	S3—O23	1.5618 (18)
Cd3—O1W	2.2272 (19)	S4—O24	1.5189 (19)
Cd3—O3W	2.278 (2)	S4—O14	1.5435 (18)
Cd3—O31	2.3201 (17)	S4—O34	1.5544 (18)
			
O13—Cd1—O32^i^	95.70 (7)	O34—Cd2—O21	68.10 (6)
O13—Cd1—O22	96.10 (7)	O14^ii^—Cd2—O21	134.22 (6)
O32^i^—Cd1—O22	135.43 (6)	O33—Cd2—O21	89.52 (6)
O13—Cd1—O21	92.82 (6)	O11—Cd2—O21	54.99 (5)
O32^i^—Cd1—O21	136.46 (6)	O24^ii^—Cd2—O21	148.24 (6)
O22—Cd1—O21	85.53 (6)	O23—Cd2—O21	74.20 (6)
O13—Cd1—O12	147.85 (6)	O14—Cd2—O21	119.61 (5)
O32^i^—Cd1—O12	89.36 (6)	O2W—Cd3—O1W	85.69 (8)
O22—Cd1—O12	59.93 (6)	O2W—Cd3—O3W	173.70 (9)
O21—Cd1—O12	105.18 (6)	O1W—Cd3—O3W	92.38 (8)
O13—Cd1—O31	138.85 (6)	O2W—Cd3—O31	97.98 (8)
O32^i^—Cd1—O31	89.02 (6)	O1W—Cd3—O31	160.09 (7)
O22—Cd1—O31	108.83 (7)	O3W—Cd3—O31	85.86 (7)
O21—Cd1—O31	58.40 (6)	O2W—Cd3—O12	99.36 (8)
O12—Cd1—O31	72.77 (6)	O1W—Cd3—O12	82.53 (7)
O13—Cd1—O23	58.36 (6)	O3W—Cd3—O12	86.31 (7)
O32^i^—Cd1—O23	70.94 (6)	O31—Cd3—O12	77.57 (6)
O22—Cd1—O23	148.13 (6)	O2W—Cd3—O11^iii^	86.15 (8)
O21—Cd1—O23	77.80 (6)	O1W—Cd3—O11^iii^	104.64 (7)
O12—Cd1—O23	150.86 (6)	O3W—Cd3—O11^iii^	88.54 (7)
O31—Cd1—O23	85.29 (6)	O31—Cd3—O11^iii^	95.15 (6)
O13—Cd1—O12^i^	81.22 (6)	O12—Cd3—O11^iii^	171.36 (6)
O32^i^—Cd1—O12^i^	55.66 (6)	O34—Cd4—O4W	166.47 (7)
O22—Cd1—O12^i^	84.10 (6)	O34—Cd4—O5W	91.25 (7)
O21—Cd1—O12^i^	167.41 (6)	O4W—Cd4—O5W	82.57 (8)
O12—Cd1—O12^i^	75.58 (6)	O34—Cd4—O32^iv^	103.74 (7)
O31—Cd1—O12^i^	132.23 (5)	O4W—Cd4—O32^iv^	84.65 (7)
O23—Cd1—O12^i^	107.85 (5)	O5W—Cd4—O32^iv^	162.15 (7)
O34—Cd2—O14^ii^	93.39 (6)	O34—Cd4—O23^v^	103.74 (6)
O34—Cd2—O33	95.19 (6)	O4W—Cd4—O23^v^	88.31 (6)
O14^ii^—Cd2—O33	135.02 (6)	O5W—Cd4—O23^v^	89.67 (7)
O34—Cd2—O11	88.74 (6)	O32^iv^—Cd4—O23^v^	77.53 (6)
O14^ii^—Cd2—O11	84.47 (6)	O34—Cd4—O21	78.39 (6)
O33—Cd2—O11	139.70 (6)	O4W—Cd4—O21	90.23 (6)
O34—Cd2—O24^ii^	143.60 (6)	O5W—Cd4—O21	95.87 (7)
O14^ii^—Cd2—O24^ii^	60.23 (6)	O32^iv^—Cd4—O21	96.59 (6)
O33—Cd2—O24^ii^	88.82 (7)	O23^v^—Cd4—O21	174.04 (6)
O11—Cd2—O24^ii^	111.03 (6)	O11—S1—O21	103.67 (10)
O34—Cd2—O23	134.72 (6)	O11—S1—O31	105.04 (10)
O14^ii^—Cd2—O23	131.44 (6)	O21—S1—O31	100.83 (10)
O33—Cd2—O23	59.77 (6)	O22—S2—O32	103.91 (11)
O11—Cd2—O23	89.63 (6)	O22—S2—O12	101.66 (10)
O24^ii^—Cd2—O23	77.64 (6)	O32—S2—O12	102.03 (10)
O34—Cd2—O14	57.77 (6)	O33—S3—O13	105.97 (10)
O14^ii^—Cd2—O14	76.06 (7)	O33—S3—O23	101.25 (10)
O33—Cd2—O14	71.77 (6)	O13—S3—O23	102.63 (10)
O11—Cd2—O14	139.30 (6)	O24—S4—O14	102.06 (10)
O24^ii^—Cd2—O14	89.80 (6)	O24—S4—O34	104.78 (11)
O23—Cd2—O14	129.84 (6)	O14—S4—O34	101.50 (10)

Symmetry codes: (i) −*x*+1, −*y*, −*z*+1; (ii) −*x*, −*y*+1, −*z*+1; (iii) −*x*+1, −*y*+1, −*z*+1; (iv) −*x*+1, *y*+1/2, −*z*+3/2; (v) *x*, −*y*+1/2, *z*+1/2.

### Hydrogen-bond geometry (Å, °)

**Table:**

*D*—H···*A*	*D*—H	H···*A*	*D*···*A*	*D*—H···*A*
O1W—H1WA···O13^i^	0.82 (3)	1.88 (2)	2.693 (3)	170 (4)
O1W—H1WB···O14^vi^	0.82 (3)	1.93 (2)	2.679 (3)	151 (3)
O2W—H2WA···O4W^iv^	0.82 (3)	1.93 (2)	2.719 (3)	162 (4)
O2W—H2WB···O6W	0.82 (3)	1.95 (2)	2.681 (3)	149 (4)
O3W—H3WA···O6W^vii^	0.82 (3)	2.02 (2)	2.833 (3)	172 (4)
O3W—H3WB···O22^i^	0.82 (3)	2.29 (2)	3.092 (3)	168 (4)
O4W—H4WA···O22	0.82 (3)	1.87 (2)	2.651 (3)	159 (3)
O4W—H4WB···O31^v^	0.82 (3)	1.98 (2)	2.780 (3)	167 (3)
O5W—H5WA···O33	0.82 (3)	2.05 (2)	2.848 (3)	167 (4)
O5W—H5WB···O24^viii^	0.82 (3)	1.92 (2)	2.708 (3)	162 (4)
O6W—H6WA···O24^ix^	0.82 (3)	2.16 (2)	2.876 (3)	146 (4)
O6W—H6WB···O33^x^	0.82 (3)	2.18 (2)	2.948 (3)	157 (4)

Symmetry codes: (i) −*x*+1, −*y*, −*z*+1; (vi) *x*+1, *y*, *z*; (iv) −*x*+1, *y*+1/2, −*z*+3/2; (vii) *x*, −*y*+1/2, *z*−1/2; (v) *x*, −*y*+1/2, *z*+1/2; (viii) −*x*, *y*−1/2, −*z*+3/2; (ix) −*x*+1, *y*−1/2, −*z*+3/2; (x) *x*+1, −*y*+1/2, *z*+1/2.
